# Emerging Understanding of Gut Microbiome in Colorectal Cancer and Food-Related Intervention Strategies

**DOI:** 10.3390/foods14173040

**Published:** 2025-08-29

**Authors:** Jie Zhang, Zhao-Jun Wei, Guangsen Fan

**Affiliations:** 1 School of Food and Health, Beijing Technology and Business University, Beijing 100048, China; 17354278037@163.com; 2 School of Food and Biological Engineering, Hefei University of Technology, Hefei 230009, China; 3 School of Biological Science and Engineering, North Minzu University, Yinchuan 750021, China

**Keywords:** colorectal cancer (CRC), gut microbiota, *Fusobacterium nucleatum*, enterotoxigenic *Bacteroides fragilis*, inflammation, fecal microbiota transplantation (FMT)

## Abstract

Colorectal cancer (CRC) is one of the most common cancers, accounting for approximately 10% of all new cancer cases globally. An increasing number of studies have revealed that the gut microbiome is strongly associated with the pathogenesis and progression of CRC. Based on these advances, this review delineates the mechanistic links between specific microbes and CRC, as well as emerging food-related nutritional intervention strategies. In vivo and in vitro studies have pinpointed the implications of key microbes such as *Fusobacterium nucleatum*, certain strains of *Escherichia coli*, enterotoxigenic *Bacteroides fragilis*, and *Enterococcus faecalis*, among others, and metabolite involvement and immune responses. Particular attention is paid to the roles of intratumoral microbiota in the development and treatment of CRC, given their direct interaction with tumor cells. Various food-related nutritional intervention strategies have been developed to mitigate CRC risk, including probiotics, antibiotics, or the administration of bioactive compounds such as luteoloside. Finally, we outline critical research directions regarding the influence of animal lineage, carcinoma location, population demographics, the application of advanced in vitro models, and the mediatory roles of gut-associated epithelial cells. In summary, this review might consolidate our current knowledge on the contribution of gut microbiota to CRC and highlights the microbe-based strategies to enhance nutritional interventions for this disease.

## 1. Introduction

Colorectal cancer (CRC) stands among the leading malignancies and ranks as the third-most prevalent cancer worldwide [1,2,3,4,5,6]. Extensive epidemiological surveillance indicates that the incidence of early-onset CRC has been increasing across many countries in recent decades. Notably, rises in early-onset CRC have been well documented in the USA, New Zealand, and various European nations, representing a significant health concern and burden, particularly among young adults [7,8,9]. As a multifaceted intestinal disorder, CRC development is multifactorial, involving intricate interactions between genetic and environmental factors. Several hereditary syndromes associated with CRC are well characterized, such as Lynch syndrome and familial adenomatous polyposis. However, hereditary factors account for only a minority of CRC cases, approximately 12%–35% based on twin and family studies [10,11,12]. This relatively low heritability underscores the importance of environmental influences, especially in the etiology of sporadic CRC.

Among environment-related cues, dietary patterns are well recognized in the pathogenesis of CRC, with a particular emphasis on the heightened risk associated with a Western diet [7,13]. This correlation was further supported by a prospective cohort followed for 32 years and comprising 17,217 men and women [14], which revealed that a Western diet was linked to a higher incidence of CRC, with a relative risk of 1.31 (95% CI, 1.15–1.48). Since diet shapes the intestinal microbial ecosystem in various ways, the gut microbiome is increasingly regarded as a key factor in CRC development [15]. This research trend aligns with the growing appreciation of the roles that microorganisms play in tumor biology.

The gut microbiome is a densely populated ecosystem comprising approximately 10^13^–10^14^ microorganisms occupying the gastrointestinal (GI) tract of humans and animals [16,17,18,19]. This microbial community is predominantly composed of Firmicutes or Bacteroides, which in sum account for about 90% of the total bacterial population [20]. Accumulating evidence suggests that the gut microbiome plays a crucial role in shaping brain development and activity. Cross talk between symbiotic microbes and the colorectal epithelium provides a fundamental physiological context for the cellular dynamics of intestinal tissues, while dysbiosis-led inflammation has long been recognized as a primary factor in carcinogenesis [15,21]. Of particular interest is the relationship between the gut microbiome and CRC, supported by a variety of reports [22,23,24,25]. This association is further cemented by the fact that microbiome compositions vary significantly depending on anatomical sites, whereas the vast quantity of the large-bowel microbiome may potentiate their mechanistic links to CRC development more than carcinogenesis at other locations. Consequently, microbiome-based approaches are emerging as valuable tools for the prevention, diagnosis, and treatment of CRC.

## 2. Associations Between Gut Microbiome and CRC

The gut microbiome exerts a decisive influence on both the development and progression of CRC. In a specific sense, a series of bacterial strains are able to promote carcinogenesis, while others show protective functions. It is critical to incorporate both plasma- and fecal-related measures into the diagnosis of CRC [26]. Herein, we outline the key bacterial species and summarize the possible mechanisms underlying their links to CRC development (Table 1).

### 2.1. Fusobacterium Nucleatum Is a Major Risk Factor of CRC

*Fusobacterium nucleatum* (*F. nucleatum)* is an anerobic commensal and pathogen associated with a wide range of human conditions, such as adverse pregnancy outcomes, gastrointestinal disorders, and even neurological illnesses. FadA adhesion/invasion conserved in this bacterium is normally regarded as a key virulence factor for *F. nucleatum*-related diseases [27]. *F. nucleatum* was originally identified from sequencing-based studies of human CRC samples [1,28], with its roles empirically demonstrated at multiple stages of CRC biology. As a consequence, *F. nucleatum* has become the best-defined CRC-related microbe in the human intestine. In population correlation studies, meta-analyses across multiple cohorts have shown that *F. nucleatum* is significantly enriched in cancer tissues. In vitro mechanism studies have shown that *F. nucleatum* adsorbs Gal-GalNAc through the Fap2 protein, inhibits T-cell immune response, and activates the Wnt/β-catenin pathway to promote proliferation. An in vivo intervention model of *F. nucleatum* indicates that it promotes tumor growth in human-derived tumor xenograft (PDX) models and increases tumor numbers in APC^Min/+^ mice.

There is a major limitation pertaining to relevant studies: while cohort studies demonstrate significant correlations between CRC and gut microbiota, causative evidence is relatively scarce for PDX/organoid models and animal interventions. More causative proof is anticipated to assist in the better elaboration of this important pathogenesis.

While *F. nucleatum* represents the most mechanistically grounded microbial contributor to CRC, its precise etiological roles require further refinement. Future research should prioritize human-relevant model systems, strain-level analyses, and combinatorial microbial ecology to translate associative observations into validated causal mechanisms for therapeutic targeting.

A Chinese prospective cohort [29] consisting of 101 CRC patients revealed that the median abundance of *F. nucleatum* in tumor tissues was markedly higher than in samples from normal patients, being overrepresented in 87.1% of CRC samples (88/101). This is another piece of proof that *F. nucleatum* in CRC requires close attention. Of note, a progressive trend of elevated abundance of *F. nucleatum* was observed in CRC, implying considerable progressive relevance throughout the developmental stage [30]. The activating functionality of *F. nucleatum* further showed the extent of selectivity, where it was associated only in the proliferation of cancer cells, but not in noncancerous cell lines [31].

From a mechanistic perspective, the association between *F. nucleatum* and CRC is widely attributed to the gene function of FadA. As an adhesin, FadA enables *F. nucleatum* to invade host tissues and induce oncogenic responses that stimulate tumor growth. Specifically, FadA facilitates the activation of β-catenin signaling through its binding to E-cadherin, which also triggers an inflammatory response that further supports oncogenic progression [31]. In addition to Wnt/β-catenin signaling, a collection of immune cells or proteins can also be activated by the prevalent existence of *F. nucleatum* in colons. For instance, CD3+ T-cell density was inversely correlated with the abundance of *F. nucleatum* [32], which stimulated tumor-promoting cytokines including IL-17 and TNF-α (tumor necrosis factor-α) through NF-κB signaling [33]. *F. nucleatum* can also influence the function of tumor-infiltrating lymphocytes and natural killer cells through collaboration with its molecular partner Fap2. A potential receptor for Fap2-mediated binding is Gal-GalNAc (galactose *N*-acetyl-D-galactosamine), which is commonly expressed at high levels in many tumor cell types [1,34].

**Table 1:** Microbial relevance in CRC development.

Microbes	Organisms	CRC-Modulating Effects	Proposed Mechanisms	References	Correlations	Models
*F. nucleatum*	Frozen tumor specimens from humans	Positive correlation with lymph node metastasis	ND	[28]	Association	Human
	Human samples and Apc ^(Min/+)^ mouse	Potentiates the intestinal tumorigenesis	Recruitment of tumor-infiltrating myeloid cells	[35]	Association and interventional	Human and animal
	Chinese cohorts	Enriched in CRC tissues and associated with CRC development and metastasis	ND	[29]	Association	Human
	A large cohort of 616 participants	Elevated abundance from intramucosal carcinoma to more advanced stage	ND	[30]	Association	Human
	Cell line and humans	Stimulates growth of CRC cells	FadA binds to E-cadherin, activates β-catenin signaling, and differentially regulates the inflammatory and oncogenic responses	[31]	Interventional and association	In Vitro and human
	598 rectal and colon carcinoma cases	Overrepresented in tumor samples	*F. nucleatum* is inversely associated with CD3+ T-cell density in colorectal carcinoma tissue	[32]	Association	Human
	A Mexican cohort	Enriched in CRC tissues	ND	[33]	Association	Human
Enterotoxigenic *Bacteroides fragilis*	Colonic mucosa of patients with familial adenomatous polyposis	Associated with faster tumor onset and greater mortality	Increased interleukin-17 in the colon and DNA damage in colonic epithelium	[36]	Association	Human
	Nude mice	Promoted colorectal carcinogenesis	Upregulated JMJD2B levels in a TLR4-NFAT5-dependent pathway	[37]	Interventional	Animal
	40 CRC patients	KRAS mutations positively correlate with the abundance of this bacterium	Existence of this bacterium affected by miR3655/SURF6/IRF7/IFNβ axis	[38]	Association	Human
	ETBF-treated cells and patients	Promotes intestinal inflammation and malignancy	Inhibiting exosome-packaged miR-149-3p	[39]	Interventional and association	In vitro and human
	Mice	Induces colitis, colonic hyperplasia, and tumor formation	Activation of T helper type 17 T-cell responses	[40]	Interventional	Animal
	Apc^Min^ mice	Induces onset of distal colon tumorigenesis	IL-17-dependent NF-κB activation, which relays CXCR2-expressing polymorphonuclear immature myeloid cells in a mucosal Th17 response	[41]	Interventional	Animal
pks+ *E. coli*	147 Caucasian cohort	*Escherichia coli* was enriched in carcinoma samples compared with both healthy and advanced adenoma sample	ND	[42]	Association	Human
	3741 stool metagenomes from 18 cohorts for cross-stage	Although not significant, the carriage of colibactin-producing genes by *E. coli* and *Klebsiella* spp. was increased in CRC; however, no correct time point for an impact of pks+ *E coli* was captured on CRC progression	ND	[43]	Association	Human
	Human intestinal organoids and two separate cohorts	Mutational signature in colorectal cancer was detected via colonization of pks+ *E. coli*	Presumptively, alkylate DNA on adenine residues induced double-strand breaks in cultured cells	[44]	Association	Human
*Streptococcus*	Human epithelial colonic Caco-2 cells and rat colonic mucosa	*S. bovis* proteins showed procarcinogenic properties	Promoted release of CXC chemokines and prostaglandin E2, correlated with in vitro overexpression of COX-2	[45]	Interventional	In vitro and animal
	50 colorectal cancer, 14 colorectal adenoma patients, and controls	Higher levels of serum *S. gallolyticus* IgG antibodies were associated with adenoma patients	NF-kappa B and IL-8 mRNAs more highly expressed in tumorous sections	[46]	Association	Human
	Cancer cell line and mouse models	Proliferation-promoting strains can promote carcinogenesis, while others cannot	ND	[47]	Interventional	In vitro and animal
*Peptostreptococcus anaerobius*	Mice and patients with CRC in Hong Kong	Contributes to colon cancer formation	*P. anaerobius* interacted with TLR2 and TLR4 to increase intracellular levels of reactive oxidative species, promoting cholesterol synthesis and cell proliferation	[48]	Interventional and association	Animal and human
	Apc^Min/+^ mice and cell line	Adhere to the CRC mucosa and accelerate CRC development	Via a PCWBR2-integrin α2/β1-PI3K-Akt-NF-κB signaling axis	[49]	Interventional	Animal and in vitro
*Salmonella*	Animal model	Enhances colonic tumorigenesis	*Salmonella* protein AvrA upregulated transcriptional activity of STAT3 and its target genes	[50]	Interventional	Animal
	2D and organotypic 3D cultures	Promoting a microenvironment conducive to malignant transformation together with the loss of APC (adenomatous polyposis coli)	Reduced DNA repair capacity and inability to activate adequate checkpoint responses, as well as increased genomic instability	[51]	Interventional	In vitro
	Mouse models	Disrupts tumors	This therapy was accompanied by a compromised activation of tumor infiltrating lymphocytes	[52]	Interventional	Animal
Fungi and virus	Cohort patients from Hong Kong	Abundance of 14 fungal biomarkers distinguished CRC from controls, characterized by a higher Basidiomycota–Ascomycota ratio	ND	[53]	Association	Human
	74 patients with CRC in Hong Kong	22 viral taxa discriminate controls from patients	ND	[54]	Association	Human
	12 CRC patients before and after surgery	Elevated viral correlations and network connectivity were observed in CRC, exhibiting cross-kingdom correlation	ND	[55]	Association	Human
	One patient with 313-day prolonged response and three non-responders	A rapid decrease in circulating DNA with virotherapy and immunotherapy	A progressive increase in CD4+ T cells, CD8+ T cells, and B cells, along with upregulated transcriptional factors for T-cell activation	[56]	Association	Human

ND, not determined. Relevant measurements were not studied or mentioned in these references.

### 2.2. Enterotoxigenic Bacteroides fragilis and CRC

Enterotoxigenic *Bacteroides fragilis* (ETBF) is a long-studied gastrointestinal pathogen that causes inflammation and diarrhea [1]. ETBF stimulates colorectal carcinogenesis in animal models, and is also detected in the human samples of biofilms coating CRCs and adenomas, a type of precancerous colonic lesions [57,58]. This is a significant finding due to the fact that biofilm can potentiate the stress-related survival of bacterium, thereby becoming a potent driver of tumor formation. A cohort study showed that patients with a medical history of ETBF infection were at a higher risk of developing CRC [59]. This survey also observed an elevated abundance of certain *Streptococcus*, *Fusobacterium*, and *Peptostreptococcus* species in the intestinal microbiota, suggesting a potentially synergistic interplay. Multiple sequencing studies have confirmed a strong correlation among *F. nucleatum,* ETBF, and genotoxic *Escherichia coli* (pks+ *E. coli*). A proposed theory suggests that ETEF plays fundamental roles by inducing inflammation, which creates a favorable environment for colonization by pks+ *E. coli*. Subsequently, once oncogenic mutations are initiated, *F. nucleatum* is recruited to colonize the lesion site, contributing to CRC progression [60]. While more compelling evidence is needed to fully validate this theory, studies in mouse models have demonstrated a synergistic action between ETBF and pks+ *E. coli*. Co-administration of these two strains led to increased tumor risk compared with mono-colonization. This synergy might originate from the distinctive and interconnected functions of ETBF and pks+ *E. coli*: ETBF promotes mucin degradation, while pks+ *E. coli* exhibits genotoxic effects [36,61].

Liu et al. [37] discovered that epigenetic elements are implicated in the ETBF-mediated promotion of CRC. A key modification of histone H3, H3K9me3 (histone H3 lysine 9 trimethylation) was significantly reduced following ETBF infection via enhanced activity of JMJD2B (JmjC domain-containing histone demethylase 2B). This epigenetic shift underlies the ETBF-induced stemness of CRC cells. Apart from histone modifications, microRNAs (miRNAs) also played a pivotal role in this microbe-led carcinogenesis. For instance, KRAS (Kirsten rat sarcoma) affected the colonization of ETBF in CRC through a molecular relay initiated by the inhibitory activity of miR3655 [38]. This regulation was consolidated by findings that ETBF promoted CRC cell proliferation by downregulating miR-149-3p, a miRNA variant released in exosomes and responsible for regulating Th17 (T helper type 17) cell differentiation. An epidemiological survey further substantiated this association by comparing profiles in CRC patients with healthy controls [39].

ETBF exhibits immunoregulatory activities, notably promoting the activation of STAT3 (signal transducer and activator of transcription 3). The invasion of ETBF, in conjunction with NF-κB signaling, synergistically modulates the Th17 immune response during colon tumorigenesis. This proinflammatory event consequently contributes to the initiation and development of CRC [40,41]. These studies underscore the importance of Th17 response in CRC cell proliferation, representing a common mechanistic pathway shared by both *F. nucleatum* and ETBF.

In population studies, ETBF is associated with IBD and CRC risk. In in vitro mechanism studies, ETBF secretes B. fragilis toxin (BFT) cleaving E-cadherin, activates STAT3 and β-catenin signaling, and induces IL-17-dependent inflammation. In vivo intervention models showed that ETBF promotes tumorigenesis in APC^Min/+^ and multiple intestinal neoplasia (MIN) models and drove colitis-associated cancer in azoxymethane (AOM)/DSS models.

### 2.3. Enterococcus faecalis Strains Are Potential Biomarkers of CRC

*Enterococcus faecalis* (*E. faecalis*) is a Gram-positive, facultatively anerobic bacterium that colonizes the human gut early in life. It is normally considered a resilient microbe, playing neutral or even beneficial roles in human health. However, the plasticity of this species is also characterized by the presence of strains with virulence traits posing potential threats to host health [62]. Certain *E. faecalis* strains produce a kind of superoxide from membrane-associated demethylmenaquinone, which increases the risk of colorectal adenomas or CRC, probably by triggering DNA damage in intestinal epithelial cells, according to a prospective case-cohort study [63]. In addition, multiple studies [64,65] have pinpointed roles of *E. faecalis* in producing potent mutagens such as hydroxyl radicals, which contribute to DNA breaks, point mutations, and other genetic mutations related to CRC risk.

In addition to mutagenesis, a screening endeavor unraveled a key metabolite, biliverdin, which was later demonstrated to promote angiogenesis in CRC via regulation of the PI3K/AKT/mTOR signaling pathway. This CRC-modulatory activity of biliverdin was then further investigated in both in vivo and in vitro models [66]. Despite these pieces of evidence, it remains controversial whether the link of *E. faecalis* with CRC is totally verified, a situation in direct contrast to the established harmful roles of *F. nucleatum* and ETBF [67]. Additionally, the presumptive protective role of some strains further complicates the current understandings of this mechanistic correlation, pointing to the importance of clarifying the strain-specific effects.

### 2.4. pks+ E. coli Contributes to the Development of CRC

*Escherichia coli* (*E. coli*) expressing the genomic island polyketide synthase (pks+) has been proposed as a major contributor to carcinogenesis [68]. Several cohort studies and meta-analyses have observed the distinct pks+ *E. coli*-related signature in CRC patients [30,42], despite the fact that a pooled analysis of 3741 stool metagenomes did not clarify the precise developmental stage responsive to the impact of pks+ *E. coli* [43].

pks+ *E. coli* produces the genotoxin colibactin, accounting for the DNA alkylation and formation of DNA adducts in colonic epithelial cells. The abnormal status of host–microbe interplay is suggested to trigger carcinogenesis [1,68]. Another genotoxin and cyclomodulin, CIF (cycle-inhibiting factor), is also overrepresented by colonization of pks+ *E. coli*, an incident capable of blocking mitosis independently of DNA damage in normal cell lines, as well as inducing chromosomal aberrances and genomic instability in colonocytes. Of interest, CIF promotes tumor formation either alone or in conjunction with ETBF, implying a potential synergistic action [36,61,69]. When human intestinal organoids were exposed repeatedly to pks+ *E. coli* for five months, a distinct mutational signature was found in two independent cohorts with CRC [44]. This provides decisive evidence that oncogenic activity of pks+ *E. coli* is primarily attributable to its mutagenicity.

In population studies, the detection rate of *pks+ E. coli* is higher in patients with colorectal cancer (CRC) and is associated with mucosal invasion. In in vitro mechanism studies, *pks+ E. coli* synthesized the genotoxin colibactin, which led to DNA double-strand breaks and induced cellular senescence and mutation. In an in vivo intervention model, *pks+ E. coli* accelerates tumorigenesis in IL-10^−/−^ and APC^Min/+^ mice and increases tumor burden in transplanted tumor models.

### 2.5. Streptococcus Is a Potential Contributor to CRC

*Streptococcus bovis* (*S. bovis*) has been reported as a risk factor for CRC. The occurrence of *S. bovis*-induced bacteremia is an early sign for CRC oncogenesis [2,70]. *S. bovis* proteins exhibit proinflammatory properties involving IL-1 and IL-8, which may actively contribute to CRC development [45].

However, despite emerging evidence, a systematic review and meta-analysis did not confirm the clinical significance of *S. bovis* infection in CRC. On the contrary, a subspecies, *S. bovis* biotype I (*Streptococcus gallolyticus*), has an unambiguous association with cancer [71], significantly influencing the tumor microenvironment. *S. gallolyticus* is associated with proinflammatory states with the prominent signature of high levels of NK-κB and IL-8. Nonetheless, caution is warranted when interpreting these effects, as some subspecies were unable to promote host cell proliferation [46,47]. It is noteworthy that some *Streptococcus* species are dominant members of the oral microbiota. Given the associations between CRC and *Streptococcus,* future research should extend beyond fecal samples to seek microbe-related factors in CRC development.

### 2.6. Peptostreptococcus Anaerobius Is a Potential Contributor to CRC

*Peptostreptococcus anaerobius* (*P. anaerobius*) is a Gram-positive anaerobic bacterium in GI (gastrointestinal) tract. Although usually harmless, it shows an increased trend in CRC development in patients. Tsoi et al. [48] found that *P. anaerobius* was highly enriched in CRC-related stool samples, promoting proliferation of colon cells via TLR2/4-mediated ROS (reactive oxygen species) accumulation. This strain can also increase the expression of proinflammatory cytokines and recruit tumor-infiltrating immune cells, aiding in establishing a microenvironment favorable for tumor initiation [49].

In addition to its influence in CRC development, *P. anaerobius* is capable of diminishing the efficacy of anti-PD1 therapy in mouse models of CRC. Researchers [72] have leveraged myeloid-derived suppressor cells (MDSCs) to test *P. anaerobius*’s ability to impose immunosuppressive effects and curb the effective T-cell response. This adverse outcome was achieved via the activation of integrin α_2_β_1_-NF-κB signaling in cancer cells accompanied by the reinforced secretion of CXCL1. As a result, CXCR2^+^ MDSCs were recruited into tumors to exert their anticancer functions. In summary, *P. anaerobius* strengthens the resistance of CRC cells towards anti-PD1 therapy.

### 2.7. Salmonella Has Relevance to CRC

*Salmonella* is a genus of prevalent foodborne pathogens that impose a wide spectrum of threats to human health. *Salmonella* infection can cause dysbiosis in GI tracts, prioritizing the occurrence of a series of gastrointestinal diseases, including cancers [2]. A comprehensive systematic review of forty-five studies revealed that *Salmonella* is among the nine fecal microbes that are consistently associated with colorectal neoplasia [73], shedding light on the establishment of prediction models. The carcinogenic activity of *Salmonella* relies on the activation of Wnt and STAT3 signaling pathways in tumor cells, wherein AvrA proteins are substantially implicated [50]. *Salmonella* has an impact on host physiology mainly through the generation of toxins, including a genotoxin called typhoid toxin. This substance has the ability to damage DNA via the PI3K (phosphoinositide 3-kinase) pathway, an indispensable molecular event leading to oncogenesis [51].

Intriguingly, *Salmonella* can also be used in cancer therapy, offering essential tools for bacterial cancer therapy (BCT). This application adds another layer of complexity to the relationship between *Salmonella* and CRC [52]. BCT has a long history of application dating back to late 19th century, during which time *Streptococcus* was used to induce tumor regression in operable patients [74]. However, it is only recently that increasing attention has been directed toward using bacterial approaches to inhibit cancer cell proliferation, often coupled with immune-related treatment [52]. *Salmonella enterica Typhimurium* (STm) is a well-established bacterial vector due to its advantages in genetic modification and metabolic adaptation. Although colonic tumor-infiltrating lymphocytes (TILs) exhibit a variety of activation defects including decoupled IFN-γ production, this deficit could be partly overcome by restoring the expression of a master metabolic controller, c-Myc, which reflects an intricate paradox with BCT induced by STm.

### 2.8. Fungi and Viruses Are Emerging Contributors to CRC

Apart from bacteria, fungi and viruses in the gut microbiome are also involved in CRC development. The gut-related fungal community is composed of the genera *Aspergillus*, *Candida*, *Debaryomyces*, *Saccharomyces*, etc., which gained increasing attention due to their essential and distinctive roles in dictating host–microbe relations [75], with a focus on their influence on IBD (inflammatory bowel disease) and CRC. Beyond the direct interaction, fungi can collaborate with other intestinal microorganisms, posing an indirect effect on related diseases [76]. Fecal shotgun metagenomic sequencing [53] of 184 patient revealed separate fungal clusters for CRC and controls. Specifically, the abundance of 14 fungal biomarkers characterized CRC patients, including *Aspergillus flavus*, *Kwoniella mangrovensis*, *Pseudogymnoascus* sp. VKMF-4518, etc. Collectively, these fungal markers classified CRC with an AUC (area under the curve) of 0.93, suggesting significant power to predict cancer outcome. Aan important aspect that cannot be overlooked is the co-occurrence between fungi and bacteria, a network potentially playing pivotal roles in CRC development.

In terms of gut-related viruses, random forest modeling revealed a tight association between virome compositions and colorectal cancer. The cancer-associated virome consisted of temperate bacteriophages, indicating the significance of virus–bacterium interactions [77]. Another cohort study assayed 74 patients and 92 healthy controls, showing that an altered enteric virome profile with 22 viral taxa discriminated the pair of groups. In this case, the AUC reached 0.80, underscoring the scientific significance of this unique cluster of viruses [54]. Considering mechanistic insight, it is commonly known that oncoviruses act as carcinogenetic drivers through introducing DNA mutations. This pathway is saliently represented by cancers outside of GI tracts, e.g., HBV (hepatitis B virus) and human papillomavirus cause host cells to transform to malignancy by integrating the viral genome into the host genome [78]. After surgery, the microbial signatures of elevated viral correlations and network connectivity were observed to persist in a microbiome dataset collected at various time points [55].

Viruses have a flip side with respect to their impact on CRC. In recent years, virotherapy has gained growing awareness by applying oncolytic viruses (OVs) to cancer cells, as they are considered to propel direct oncolysis and cancer immune effects. Antitumor effects of OVs were consolidated by robust results from clinical trials [79]. Combined with immunotherapy, the effectiveness of oncolytic virotherapy can be considerably cemented, resulting in a progressive increase in CD4+ T cells, CD8+ T cells, and B cells in CRC patients [56]. This finding provides a promising perspective for the joint application of antitumor strategies.

### 2.9. Intratumoral Microbiota Is an Emerging Field to Impact CRC Pathogenesis

Gut microbiota can not only affect the tumor microenvironment indirectly through their metabolites or immunoregulatory activities but also affect the composition and function of the intratumoral microbiota, which displays a direct influence on tumor proliferation and inhibition [78]. Intratumoral microorganisms have been found in at least 33 major cancer types, forming a combination of context-specific microbiota. The intratumoral microbiota is an integral part of the tumor microenvironment, significantly affecting the occurrence, development, and treatment of all types of tumors. When injecting *F. nucleatum* into subcutaneous tumors, this intratumoral bacterium then modulates host Wnt signaling by binding FadA to E-cadherin, which eventually promotes tumor growth [31]. Based on its significance, the intratumoral microbiota has been viewed as a new frontier in cancer development and therapy [80], though with some significant limitations, such as laboratory contamination, low microbial load in tissues, and lack of DNA/RNA isolation standards.

The intratumoral microbiota is able to affect CRC through a series of events such as DNA mutations, carcinogenic pathways, and chronic inflammation [78]. For example, *Staphylococcus, Lactobacillus*, and *Streptococcus* were enriched in cancer cells and acted by reshaping the cytoskeleton to assist in the resistance of tumor cells against the mechanical stress in blood tumors, eventually promoting metastasis. This process is independent of the impact of the intestinal microbiota or immune system, as demonstrated by germ-free mice or immunodeficient mice [81]. Another single-cell RNA-sequencing project comprehensively checked the cell-associated bacteria within a diverse range of microniches of cancer cells. As unveiled by data, various transcriptional pathways were altered due to direct contact of intratumoral bacteria, as highlighted by inflammation, metastasis, cell dormancy, and DNA repair. Bacterial infection in cancer cells triggers a reply of translocation events in the cellular dimension, ultimately recruiting myeloid cells to bacterial regions. This study concludes the significance of the intratumoral microbiota in the constitution of the tumor microenvironment [82].

However, far from being an outright villain, the intratumoral microbiota behaves like a two-sided blade. Although the intratumoral microbiota can accelerate neoplastic expansion, it also plays roles in inhibiting tumor progression. Distinct sets of microbial profiles were identified within the tumor microenvironment of patients with long-term survival, a paradigm serving as a predictive biomarker for good-prognosis patients [83]. Some bacteria were reduced in tumor tissues compared to normal controls, as exemplified by the *Cladosporium* abundance in an analysis between tumor and adjacent breast tissues [84]. This dichotomy is also applied to its interplay with the antitumor immunity. The intratumoral microbiota is either positively or inversely linked to antitumor immune response, causing divergent cancer-related outcomes. Mechanistically, distinct molecular pathways account for the respective responsiveness: STING signaling activation and T- and NK-cell activation contribute to the potentiated antitumor efficacy, while upregulation of ROS, T-cell inactivation, and immunosuppression are associated with an attenuated antitumoral practice [78].

The investigation of the intratumoral microbiota represents a transformative approach in oncology; however, the validity of findings is intrinsically linked to overcoming substantial methodological hurdles inherent to low-biomass microbial analysis. The reliability of observations is contingent upon implementing systematic quality assurance measures to address contamination and analytical biases. Microbial DNA from reagents, kits, laboratory environments, and personnel can overwhelmingly dominate the signal from true low-biomass samples. Mitigation requires the use of dedicated, sterilized equipment, UV-irradiated workspaces, and most critically the routine inclusion of negative controls (no-template controls, NTCs)—extraction blanks and PCR blanks—across all batches. These controls are essential for bioinformatic decontamination. The microbial DNA in tumor tissue often constitutes a minuscule fraction (<0.01%) of the total DNA, making authentic signals difficult to distinguish from noise. Studies must report sequencing depth and incorporate positive controls (e.g., low-concentration mock microbial communities) to accurately define the limit of detection and ensure sufficient sensitivity. As this emerging field is in its infancy, no rigorous selection guidelines were employed in order to evade the omission of the meaningful findings. However, it must be noted that the shortfall of intramucosal microbiota research should include laboratory contamination, low microbial load in tissues, and lack of DNA/RNA isolation standards in future investigations and reviews.

### 2.10. Microbe-Derived Metabolites Mediate the Microbiota-Related Effects on CRC

Microbe-derived metabolites are well-established mediators between microbiota and CRC. The CRC-associated microbiome can be a source of oncometabolites. For instance, *B. fragilis* and *F. nucleatum* have been reported to produce succinate, an inducer of proinflammatory pathways [85]. *E. coli* can transform lysin into an intermediate L-2-hydroxylglutarate, a metabolite involved in epigenetic deregulation in certain cancers. Conversely, increased levels of SCFAs (short-chain fatty acids) in microbial communities are generally associated with a lower risk of CRC, counteracting tumor development [61]. Moreover, when pairing microbiome and metabolome data during CRC progression, Fu et al. [86] discovered that non-classic amino acid conjugation of the bile acid cholic acid (AA-CA) was increased in a CRC model, accompanied by concomitant changes in *Ileibacterium valens* and *Ruminococcus gnavus*. AA-CA plays a key role in intestinal stem cell growth, a potential signature of cancer progression. Bile acid’s role was then further validated in multiple models and studies [87,88]. Taken together, the gut microbiota can exert its CRC-modulatory effect through the generation of their metabolites.

### 2.11. Inflammation Is Suggested to Underlie the Microbial Effects on CRC

Chronic inflammation in the colon, a hallmark of IBD, creates an environment prone to carcinogenesis. Persistent inflammation leads to oxidative stress, DNA damage, and altered cellular repair mechanisms, promoting malignant transformation. Cumulative evidence shows that patients with long-standing IBD face a higher CRC risk, as classified by duration, extent, and severity [89]. Effective management of inflammation through medications like 5-aminosalicylates may also reduce CRC risk [90]. On the other hand, the microbiota exerts immunoregulatory implications, influencing multiple organs through the immune pathways [91]. Therefore, immune processes, particularly inflammation, are considered the critical link through gut microbiota and cancer dynamics.

**Figure 1:**
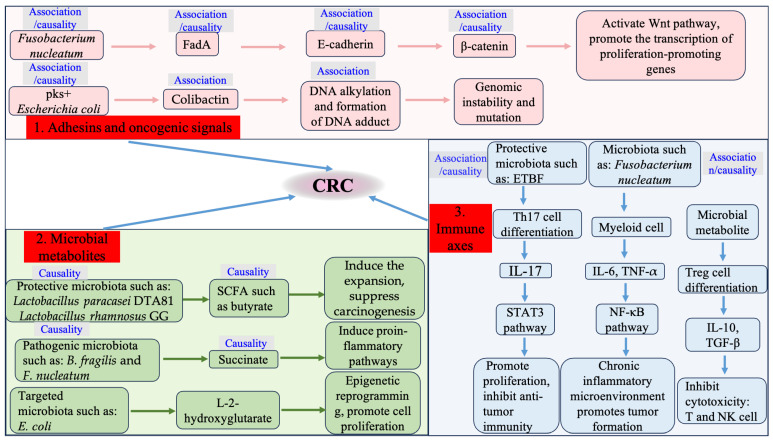
Gut microbiota-associated mechanisms involved in the pathogenesis of colorectal cancer. The gut microbiota contributes to colorectal carcinogenesis through multiple pathways, including microbial-derived factors such as metabolites and FadA/E-cadherin, activation of procarcinogenic signaling pathways such as β-catenin and PI3K/AKT/mTOR, and inflammatory pathways such as IL-1 and IL-8, CD4+ T cells, CD8+ T cells, B cells, and NF-κB.

A variety of microorganisms play similar roles in affecting IBD and CRC due to their proinflammatory or anti-inflammatory activities. A representative CRC-associated species, ETBF displays robustness in causing diarrhea and IBD [2]. This notion aligns with a previous finding that patients with IBD and CDC showed commonly reduced bacterial diversity and abundance, whereas enrichment in *F. nucleatum, E. coli* and *E. faecalis* was detected in these subpopulations compared to healthy controls [92]. This effect can be further expanded to tumor-infiltrating immune cells (TIICs), a major component of the TME (tumor microenvironment), which serves a key role in both cancer dynamics and immunotherapy [93,94]. Kikuchi et al. [95] analyzed the fresh surgically resected specimens of CRC patients, discovering that *Faecalibacterium*, *Ruminococcaceae*, *Eubacterium*, and *Bacteroides* were increased in patients with a high proportion of M1 tumor-associated macrophages. Considering lymphocytes, *Bacteroides* and *Faecalibacterium* were also found at elevated levels in patients with a high proportion of Tregs (regulatory T cells). Based on the intricate complexities in the mutual interactions among microbiota, inflammation, and CRC therapy, Cao et al. [96] recently revealed a synergistic action involving gut microbiota- and immune checkpoint inhibitor (ICI)-based immunotherapy. This study was performed at single-cell resolution, showing that intact microbiota combined with ICIs combinatorially increased the proportions of CD8+, CD4+, and γδT cells, followed by an enhanced antitumor response. In summary, the complexity of the gut microbiota and immune response provides a compelling rationale for further elucidating the specific roles of the gut microbiota within various immune contexts (Figure 1). In summary, multiple strains were suggested to correlate with CRC-related processes via metabolites and inflammatory pathways.

## 3. Emerging Intervention Strategies for CRC

Given the associations between the gut microbiota and CRC, microbiota-intervention approaches hold promise to alter CRC status. Moreover, as diet plays crucial roles in both microbiota composition and CRC development, dietary factors with microbiota-altering capacity, such as natural bioactive compounds, are known to serve as competent candidates for CRC intervention.

### 3.1. Antibiotics

Antibiotic treatment is a commonly used method to interfere with the gut microbiota, often causing dysbiosis. However, the use of specific types of antibiotics in a specific spatiotemporal context can lead to a tailored modification of specific bacterial taxa. A recent proof-of-principle study demonstrated that metronidazole could slow the growth of *F. nucleatum*-positive tumors in patient-derived xenograft models [1,97]. Given the conflicting efficacy, it is hugely controversial whether antibiotics can be used to prevent CRC in clinical practice. A meta-analysis of 4.1 million individual and over 73,550 CRC cases drew the conclusion that the pooled CRC risk was increased among individuals who used broad-spectrum antibiotics, but not for narrow-spectrum antibiotics [98]. This result gives a discriminative landscape of the antibiotic-related risks. Another consideration is cancerous lesions in CRC development and the consequential event of developing antibiotic resistance in practice [98]. Therefore, it is highly recommended that only selective antibiotics approaches be applied in efficacy tests rather than more disruptive broad-spectrum antibiotics.

### 3.2. FMT (Fecal Microbiota Transplantation)

FMT is an effective method to reshape microbiota, as well as precisely delineating the roles of microbiota instead of merely identifying several coincidental events [91]. It is rationally designed to transplant stool samples from healthy donors to patients. In particular, FMT has been considered for the treatment of a plethora of diseases, including colitis caused by antibiotic-resistant *Clostridium difficile* infection or IBD [99]. Special interest was afforded to the *Clostridium difficile* infection, and about 90% of patients were cured with this safe, inexpensive, and effective approach. On account of the close correlation between inflammatory-related conditions like IBD and CRC, numerous efforts were made to inspect the application of FMT in the prevention and treatment of CRC. In a preclinical case, FMT was piloted in a small number of CRC patients with the comorbidity of severe colitis, causing an increase in the proportion of regulatory T cells within the colonic mucosa and the attenuated colitis associated with the use of ICI (immune checkpoint inhibitor)-based immunotherapy [100]. Apart from this instance, a melanoma-associated practice also revealed that FMT could be used to overcome resistance to immunotherapy in patients who remain refractory to ICI-based treatment [101,102]. Although FMT holds significant potential, additional factors must be considered when extrapolating FMT to a broader range of clinical settings. A key challenge is the variability in microbiota transfer resistance, which is influenced by the microbiota compositions of the recipients, the microbial adaptation between donors and recipients, and other unspecified factors.

### 3.3. Probiotics

**Figure 2:**
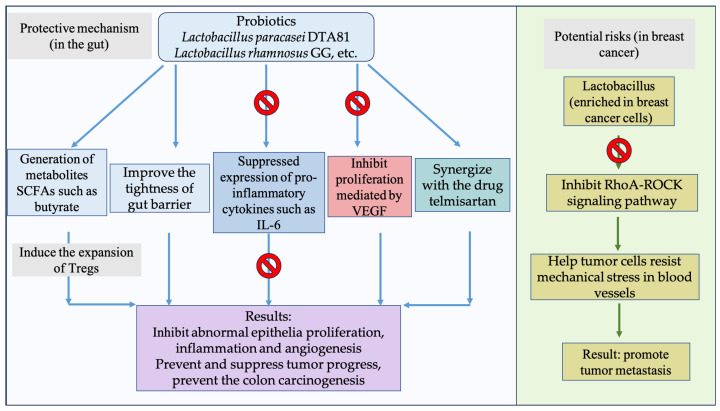
The protective mechanisms of probiotics in the intestinal environment and their potential risks in specific distant cancers. The “prohibition” symbol (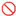) indicates suppression.

Probiotic intake is the leading strategy for deliberately reshaping gut microbiota. As the predominant genus within the category of probiotics, lactobacillus plays a multifaceted role in CRC treatment. For example, administration of *Lactobacillus paracasei* DTA81 is capable of preventing early colon carcinogenesis in male BALB/c mice [103]. *Lactobacillus rhamnosus* GG and *Bifidobacterium lactis* Bb12 have been shown to prevent abnormal epithelia proliferation and improve the tightness of the gut barrier, suppressing tumor progress in a CRC mouse model [104,105]. The coadministration of a *Lactobacillus* mixture consisting of *Lactobacillus delbrueckii* and *Lactobacillus fermentum* potentiated the antitumor effect of telmisartan in rats [106]. These strains exerted their beneficial effects primarily through the generation of metabolites such as SCFAs. A representative form of SCFA is butyrate, which is known to induce the expansion of Tregs in colorectal tissues and suppress carcinogenesis [107]. Other mechanisms involve the suppressed expression of proinflammatory cytokines such as IL-6 or the inhibited proliferation mediated by vascular endothelia growth factor (VEGF). Some lactobacillus strains augment their existence in cancer-associated tissues. Fu et al. [81] stated that *Lactobacillus* was enriched in breast cancer cells, helping tumor cells resist mechanical stress in blood vessels through inhibiting the RhoA-ROCK signaling pathway, ultimately promoting tumor metastasis. Such contradictory findings emphasize the need to define clear contexts—host, tumor subtype, and microbial strain—before deploying probiotic interventions in cancer therapy (Figure 2).

### 3.4. Dietary Intervention and CRC

It is long established that the Western diet is tightly associated with the occurrence of CRC [7,108]. A growing body of literature has attempted to ameliorate CRC-related symptoms by dietary intervention in the gut microbiota. Rodrigues et al. [109] employed black raspberries to suppress colitis and colon tumorigenesis by altering the beta diversity and relative abundance of Erysipelotrichaceae, Bifidobacteriaceae, etc. of the fecal microbiome. Papier et al. performed a diet-wide prospective analysis of CRC risk of 12,251 cases among 542,778 women in the UK. Based on the results, alcohol intake had the strongest association with cancer risk, along with the consumption of red and processed meat. Contrarily, genetically tailored milk was inversely correlated with CRC risk, in line with a relatively weaker association of breakfast cereal, fruit, wholegrain, folate, vitamin C, etc. [110], It was then concluded that dietary habits had a profound impact on tumorigenesis, with protective roles principally assigned to dairy products, a benefit probably driven by calcium intake.

### 3.5. Natural Compounds and CRC

**Figure 3:**
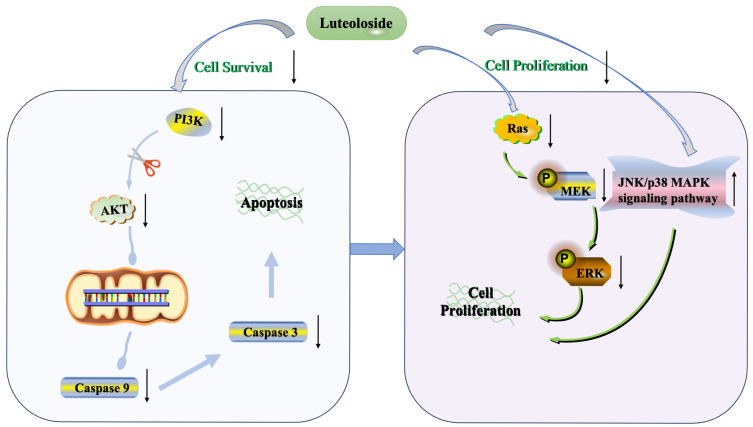
Overview of mechanisms underlying the effects of LUS on cell apoptosis and proliferation in HCT116 cells. This schematic illustrates the proposed mechanisms by which LUS exerts effects of intervention. The upward arrow indicates improvement in the relevant phenotypes; the downward arrow denotes inhibition. In summary, antibiotics, FMT, and probiotics are considered major approaches to interfere with the microbiota, thereby exerting an influence on CRC.

A constellation of natural compounds exhibit antitumor activities, highly represented by resveratrol, curcumin, betulinic acid, and other antioxidant substances with plant-based origins [111]. This review only recapitulates a limited number of natural compounds, focusing on their commonly modulated pathways. A well-established antioxidant, curcumin was found to trigger apoptosis by either inducing or inhibiting several signaling pathways, particularly suppressing anti-apoptotic proteins of the Bcl2 family amid apoptosis protein inhibitors including survivin and XIAP [112]. A similar mechanism was leveraged by luteoloside (LUS, cynaroside), a flavonoid that is isolated from several species belonging to the Apiaceae, Poaceae, etc. and possesses numerous health-promoting effects [113]. Our previous studies showed that luteoloside can induce apoptosis in colorectal cancer cells via mitochondrial and PI3K/AKT signaling pathways, wherein levels of caspase 3 and caspase 9 were significantly downregulated [114]. This finding demonstrates the pivotal role of apoptosis in the LUS-driven antitumor processes. Apart from cellular physiology, LUS also showed pronounced inhibitory activity in cell migration and invasion cells. An epigenetic interactive network, characterized by miR-6240 and migration regulator RAS/ERK/MAPK signaling, was prominently implicated in the antitumor efficacy provoked by LUS [115] (Figure 3).

## 4. Future Perspectives

The association between a specific microbe and CRC is neither uniform nor immutable [96,116,117,118]. Although *F. nucleatum* is frequently portrayed as a major gut-derived bacterium linked to CRC development, in a comprehensive study and meta-analysis recapitulated fecal microbiota of 589 patients at different CRC stages and compared findings of 15 published data [119], the authors unexpectedly found that *F. nucleatum,* among other well-established microbiome CRC targets, was not significantly associated with CRC patients when controlling for multiple covariates. This raises the critical issue to all relevant associations that all data must be carefully reevaluated before being translated into clinical applications. One of the major factors is the CRC subtype. Young-onset CRC (yoCRC) is associated with a unique microbial profile compared with average-onset CRC (aoCRC). Specifically, yoCRC tumors were enriched with *Akkermansia* and *Bacteroides*, whereas aoCRC tumors were associated with greater abundance of *Fusobacterium*, *Bacillus*, *Staphylococcus*, and *Listeria*, among others. Moreover, additional parameters such as tumor location, sidedness, stage, and obesity were also massively implicated in this process. This argument was further supported by a pooled analysis from 18 cohorts, which revealed that commensal strains had strong associations with late-stage CRC and specific gut species distinguished left-sided versus right-sided CRC [43]. Therefore, microbial data must be interpreted within a multidimensional framework that accounts for tumor subtype, patient phenotype, and methodological confounders. Only by integrating these layers of information can we reliably harness microbiome-based strategies for preventing or treating CRC.

The current experimental designs regarding either animal-based or population-based studies are normally associated with their respective drawbacks. Researchers compared the mouse lineages involved in mouse models of colitis, observing remarkable differences in various endpoint measures [120]. This issue is often overlooked, which confounds the pooled elaboration of data from different trials. Thus, it remains a challenging task to integrate data from distinct origins to draw a convincing conclusion. In terms of cohort study, the ethnicity of the studied population is often ignored, which plays crucial roles in ensuing correlation models. Of note, even after controlling for socioeconomic, dietary, or treatment covariates, race and ethnicity still display distinct effects on the microbiome composition, as demonstrated in a cohort study targeting CRC-associated patients in a multiracial community in the United States [121]. Based on these settings, efforts should be made in future to rectify related correlation data via controlling with identity information in groups of animals or humans. Moreover, a uniform and reasonable criterion should be set to integrate all the indispensable considerations in future investigations. Another essential point is the inconsistency between species- and strain-level sequencing, with new-generation sequencing technologies likely leading to new findings ignored in previous data analysis.

Emerging experimental platforms are reshaping how the microbiome–CRC axis is studied, such as cocultured organoid systems and gnotobiotic mouse models. The 3D organoid model system has garnered increasing attention in deciphering host–microbe interactions. Organoids are generated from adult and fetal tissues, embryonic stem cells, or other pluripotent stem cells, creating an interactive environment for host and microbes, which is becoming an optimal model to mimic genuine in vivo situations. The invention and development of the organoid system provides the best chance to study the influence of a specific microbial community on the CRC microenvironment without the consumption of living organisms. Particular attention has been paid to the patient-derived organoid (PODs), as the cues directly from the patient tissues could be retrieved and incorporated into the model system. Compared to the ex vivo models, the application of gnotobiotic animals still plays indispensable roles in characterizing the causal roles of a specific microbe. The germ-free mouse offers a fundamental condition, but still with physiological concerns. Many reports have shown that GF mice display changes in immune responses compared with their non-GF counterparts, as well as altering the animals’ behavioral profiles. Colonization of a specific microbe is then likely to aggravate the relevant symptoms, thereby failing to reflect on the bona fide circumstances in a CRC-associated animal model. To bridge this gap, the field is moving toward models that harbor diverse, functionally balanced microbial networks that more faithfully replicate the human gut ecosystem, thereby yielding clearer mechanistic insight from microbial status to tumor initiation and progression.

The gut-associated epithelium lies at the interface between microbe and colon tissue, with significant involvement in the oncogenesis and development of CRC. Intestinal epithelial cells (IECs) have been found to direct intestinal homeostasis by orchestrating communication between microbes and mucosal immunity in physiological and inflammatory conditions [122]. The host tissue that is in direct contact with gut-derived microbes is primarily the epithelial cells, which form a complex protein signaling network responsive to colonization or invasion by different microbial strains. Conversely, CRC originates in the epithelial layer of either right- or lift-sided CRC. Therefore, it remains an important and challenging task to precisely delineate the roles of IECs in microbe-associated CRC pathogenesis, as well as the development of nutritional and pharmacological interventions for colon-related diseases.

In summary, the most promising intervention approaches should be recognized as probiotic interventions due to GRAS status, vigor, and robustness in positively modifying the gut microbiota. Actually, numerous studies have implicated probiotics in the treatment of CRC, yielding variable promising effects, with a focus on lactobacillus strains residing in both gut and tumor tissues. In terms of a clinical translation roadmap, while great progress has been made in elucidating the contributions of gut microbiota to the diagnosis and treatment of colorectal cancer, it is still too early to accomplish clinical translation using microbiota-related measurements. The first primary step might be to select persuasive biomarkers for clinical validation, like stage-specific microbiome signatures, with predictive compositions at least up to 0.8 AUC (area under curve). *F. nucleatum* should be assigned much importance due to its broad-ranging research basis and the mounting evidence of its clinical correlations. However, it should be noted that its significance has only been considered in a specific medicinal context, like a specific animal model or fixed set of cohort populations.

Therefore, the translation of microbiome science into CRC clinical practice is a multistage process. Diagnostic biomarkers are poised for immediate validation in real-world settings. Contrarily, therapeutic interventions require meticulous mechanistic groundwork and a commitment to precision medicine. Success hinges on interdisciplinary collaboration to integrate microbial diagnostics into the existing oncology framework.

## 5. Methodology

A comprehensive computerized search was conducted across different databases, such as PubMed (http://www.ncbi.nlm.nih.gov/pubmed), Web of Science (https://mjl.clarivate.com/search results), Science Direct (https://www.sciencedirect.com/), and Google Scholar (https://scholar.google.com/). We carefully reviewed the reference lists of all significant studies and reviews. The following terms were used either singly or in combination as inclusion criteria:

1. “gut microbiota” OR the concrete terms of specific bacterium.
2. “CRC” OR “colorectal cancer.”

For clinical studies, the category “Clinical trial” was selected in the PubMed database.

## Data Availability

No new data were created or analyzed in this study.
